# Relocation of *Dodecaseta* McCammon & Stull, 1978 (Annelida, Capitellidae) in *Notodasus* Fauchald, 1972

**DOI:** 10.3897/zookeys.715.13936

**Published:** 2017-11-21

**Authors:** María E. García-Garza, Jesus A. de León-González, Leslie H. Harris

**Affiliations:** 1 Universidad Autónoma de Nuevo León, Facultad de Ciencias Biológicas, Laboratorio de Biosistemática, AP. 5 ‘‘F’’, San Nicolás de los Garza, Nuevo León, México; 2 Natural History Museum of Los Angeles County, 900 Exposition Boulevard, Los Angeles, California, 90007, U.S.A.

**Keywords:** Polychaeta, Capitellidae, *Dodecaseta*, *Notodasus*, new combinations

## Abstract

The capitellid polychaete genus *Dodecaseta* McCammon & Stull, 1978 is relocated in *Notodasus* Fauchald, 1972. Two species are redescribed based on examination of type material and three new combinations are proposed: *Notodasus oraria* (McCammon & Stull, 1978), *N. eibyejacobseni* (Green, 2002). *N. fauchaldi* (Green, 2002). *N. kristiani* (García-Garza et al., 2009), is synonymized under *N. oraria*. Some comments on *Dasybranchus lumbricoides* Grube, 1878 are included.

## Introduction

The study of capitellid polychaetes has been complex. Determination at the generic level is based mainly on the number of thoracic segments, the number of chaetigers with capillary chaetae, a mixture of capillaries and hooded hooks, complete (with notopodium and neuropodium), or incomplete (with notopodium only) first chaetiger, as well as the position of the transition between thorax and abdomen (Fauchald 1977). However, these characters have caused erroneous determinations when the organisms are in initial stages of growth since the capitellids display great morphological variability in these characters at different stages of development (Blake 2000).

Capitellids in early developmental stages have the first three segments with capillary chaetae while the following segments bear hooded hooks which will later be replaced by capillary chaetae in subsequent stages (Ewing 1984, George 1984). In addition to its simple morphological structure, and sometimes due to improper relaxation, fixation and preservation of specimens, confusion can occur in the establishment of new genera or species and misidentifying species by identifying immature stages. With this variability in the chaetal formula we may infer that some monotypic genera could be described from juvenile organism however this needs an in depth study of each case. In this work a review of a species belonging to *Dodecaseta* McCammon & Stull, 1978, confirmed that these species have morphological characters placing them in *Notodasus* Fauchald, 1972.

## Material and methods

Material from the Natural History Museum of Los Angeles County, Allan Hancock Foundation Polychaete Collection (LACM-AHF) and the Colección Poliquetológica de la Universidad Autónoma de Nuevo León (UANL). Methyl green staining was used to determine specific patterns of glandular areas. Specimens were submerged for a maximum of two minutes in a solution of methyl green in 70% ethanol and washed in several alcohol changes (Warren et al. 1994). Photographs were taken with a stereomicroscope Olympus SZ61 equipped with a digital camera Olympus C-7070. Editing of photos was performed using Adobe Photoshop CS6.

## Taxonomy

### Order Capitellidae Fauchald, 1977

#### Family Capitellidae Grube, 1862

##### Genus *Notodasus* Fauchald, 1972

###### Notodasus

Taxon classificationAnimaliaCapitellidaCapitellidae

Fauchald, 1972

Notodasus Fauchald, 1972: 246–247, Pl.51 fig. a-c; García-Garza 2009: 101; García-Garza, et. al. 2009: 810; García-Garza and de León-González 2011: 35; Magalhães and Bailey-Brock 2012: 28.
Dodecaseta McCammon & Stull, 1978: 40–43, figs 1–3; Green 2002: 311.

####### Type species.

*Notodasus magnus* Fauchald, 1972

####### Diagnosis.

Thorax with 11 chaetigers with bilimbate capillary chaetae first chaetiger biramous. First two abdominal chaetigers with bilimbate capillaries in both rami, subsequent chaetigers with hooded hooks. Lateral organs and branchiae present.

####### Remarks.

The genus *Dodecaseta* was established by Mc Cammon and Stull (1978) to include *Dodecaseta oraria*, a species from Southern California. These authors considered that *D. oraria* differed from the genus *Notodasus* by presenting the first thoracic chaetiger biramous and capillary chaetae in the first abdominal segments. In the original description of *Notodasus*, Fauchald (1972) described the genus with the first chaetiger being uniramous. García-Garza et al. (2009) reviewed the *Notodasus* genus based on the examination of type material, and they observed that the holotype *N. magnus* (type species of the genus) has the first chaetiger biramous, not uniramous as originally described.

The holotype of *D. oraria* is a small specimen of 12 mm long and 0.8 mm wide (LACM-AHF POLY 1248). McCammon and Stull (1978) included 12 specimens in the original description, but they did not include variation in body size of these specimens. Based on this discrepancy in the description (and other factors) we believe that the genus *Dodecaseta* should be synonymized with the genus *Notodasus*.

Blake (2000) had previously felt that *Dodecaseta* should be synonymized with *Notomastus* since the only known species form the Southern California Bight varied from *Notomastus* only in that it has notochaetae in the first abdominal chaetiger, and the presence of abdominal capillaries is not a considered generic character of capitellids.

Green (2002) expands the genus *Dodecaseta*, in order to describe two new species from the Andaman Sea (*D. fauchaldi* and *D. eibyejacobseni*) including the following characters, 12 and 13 chaetigers with capillary chaetae, with the last one or two transitional in appearance with expanded neuropodial lobes, and protruded lateral organs. However, the ammended diagnosis proposed by Green for *Dodecaseta* agrees with the diagnosis of the genus *Notodasus* Fauchald, 1972. Consequently, we believe that *D. fauchaldi* and *D. eibyejacobseni* should also be reassigned to the genus *Notodasus*.

###### Notodasus oraria

Taxon classificationAnimaliaCapitellidaCapitellidae

(McCammon & Stull, 1978) comb. n.

Figure 1

Dodecaseta oraria Mc Cammon & Stull, 1978: 41–43, figs 1–3.
Notodasus kristiani García-Garza et al., 2009: 809–823, figs 5A–D, 8E; García-Garza and de León-González 2011: 17–52; Magalhães and Bailey-Brock 2012: 33, figs 24a–d, 25c–d.

####### Material examined.

Type material *Dodecaseta oraria* Holotype (LACM-AHF POLY 1248); Paratype (LACM-AHF POLY 1250), Palos Verdes Península, California, USA, 30–180 m; *Notodasus kristiani* Holotype (UANL 6515), Varadero beach, Guaymas, Sonora, Mexico [27°54'04.3"N, 110°52'07.7"W], 1 m, July 01 2005, 20 Paratypes (UANL 6517), 2 Paratypes (LACM-AHF POLY 2213), 1 Paratype (MNNH 1508), 1 Paratype (ZMH POL), Varadero beach, Guaymas, Sonora, [27°54'04.3"N, 110°52'07.7"W], 1 m, July 01 2005, coll. J.A. de León-González (JALG) and M.E. García-Garza (MEG-G).

####### Additional material examined.

*Notodasus kristiani*, two specimens (ECOSUR), Municipal beach, Los Angeles Bay, Baja California, Mexico [28°56'32.3"N, 113°33'57.2"W] 1 m, May 24 1986, coll. P. Sánchez and E. Espinosa. One specimen (UANL 6515), Estero Rancho Nuevo, Santa Marina Bay, Baja California Sur, [24°19'15"N, 111°25'05"W] 3 m, June 21 1998, coll. JALG.

**Figure 1.**
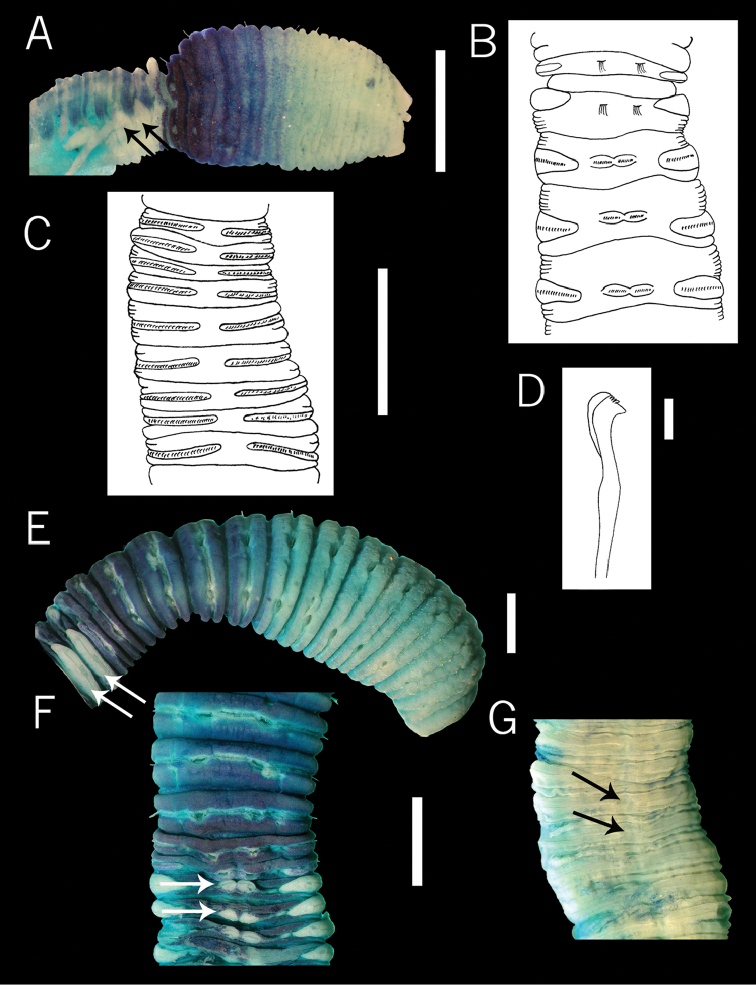
Holotypes of *Dodecaseta oraria* McCammon & Stull (**A–D**), and *Notodasus kristiani* (García-Garza et al., 2009) (**E–G**). **A** anterior end, lateral view **B** chaetiger 12-16, dorsolateral view **C** chaetigers 36-46, ventral view **D** neurohook from chaetiger 88, lateral view **E** anterior end, lateral view **F** chaetigers 9-17, dorsal view **G** chaetigers 19-27. Methyl green stain **A–C, E–G**. Scale bars: **A, B, C, E, F, G** = 1 mm, **D** = 15µm.

####### Redescription.

Holotype of *D. oraria* incomplete with 89 segments, 12 mm long, 0.8 mm wide in abdomen. Color in alcohol white-translucent. Prostomium conical with palpode. Eyespots not seen. Peristomium to the eighth thoracic chaetiger tessellated, remaining segments smooth. Thorax with 11 chaetigers, with bilimbate capillaries in both rami, first chaetiger biramous, transition between thorax and abdominal segments marked by abrupt shorting of abdominal segments. (Fig. 1A). Thoracic and abdominal segments biannulate. Notopodia lateral in first thoracic segments, in subsequent segments located dorsally. Lateral organs, between notopodia and neuropodia throughout body, those of thoracic region closer to notopodium; thoracic lateral organs larger than abdominals. Genital pores not seen. Notopodial lobes of abdominal chaetigers 3–7 fused dorsally (Fig. 1B, F), each line of hooded hooks completely separated, with around 18–20 hooks per fascicle. Neuropodial lobes project to dorsal region and separate ventrally, with chaetal fascicles with about 77–80 hooded hooks (Fig. 1A, C). Notopodial and neuropodial abdominal hooded hooks similar along body, with long anterior shaft, bulbous node, indistinct constriction, developed shoulder, short hood, posterior shaft longer than anterior one. Four rows of teeth above triangular main fang, basal row with five teeth, middle basal row with seven, middle apical row with nine and distal row with two teeth (Fig. 1D). Branchiae emerge from a ventral pore. Pygidium not seen.

Methyl green staining pattern. Peristomium and first 6 segments without staining apparently, post-chaetal part of segment 7, segments 8 to 10 and pre-chaetal part of segment 11 moderately stained, and segments 12 and 13 dark green (Fig. 1A, E).

####### Habitat.

Mud with high content of organic matter (Varadero beach), in soft sediments retained into Nastier boxes (Santa Marina bay), and mud pockets between *Mytilus edulis* beds (Los Angeles Bay, Municipal beach).

####### Distribution.

USA: Southern California Bight, Waianae outfall and Mamala Bay, Sand Island outfall, Hawaii; Mexico: Gulf of California and western coast of Baja California.

####### Remarks.

Type material of *Dodecaseta oraria* was examined and compared with type material of *Notodasus kristiani*, and we found similar morphological characters in both species: lateral notopodia in the first thoracic segments, in subsequent segments located dorsally; abdominal notopodial lobes fused dorsally (Fig. 1B, F); neuropodial lobes expanded to the dorsal side (Fig. 1A–B, E–F) and separated ventrally (Fig. 1C, G).

Based on examining type material of both species, we conclude that the specimens identified as *D. oraria* were juveniles exhibiting variation in chaetal counts, leading to a mis-identification. We conclude therefore, that *Dodecaseta oraria* is a junior synonym of *Notodasus kristiani*.

###### Notodasus fauchaldi

Taxon classificationAnimaliaCapitellidaCapitellidae

(Green, 2002) comb. n.

Figure 2

Dodecaseta fauchaldi Green, 2002: 312-313, fig. 23A–I.

####### Material examined.

Type material. *Dodecaseta fauchaldi* Paratype (LACM AHF POLY 2100) St. E-20 m/BC, North Pacific Ocean, Andaman Sea, Thailand, [8°30'N, 98°12'E] 21 m, 22 April 1996, muddy sand, coll. S. Bussarawit, Charatsee Aungtonya.

**Figure 2.**
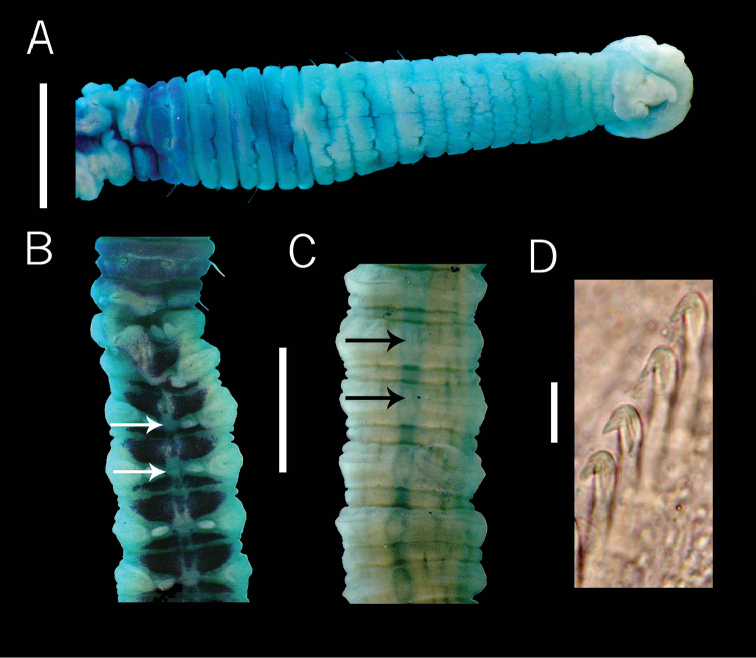
Paratype of *Dodecaseta fauchaldi* (Green, 2002). **A** anterior end, dorsal view **B** chaetigers 12-19, dorsal view **C** chaetigers 21-26, ventral view **D** neurohook of chaetiger 40, lateral view. Methyl green stain **A**. Scale bars: **A–C** = 1mm, **D** = 15 µm.

####### Redescription.

Paratype incomplete (lacking posterior end) with 44 segments, 19 mm long, 0.71 mm wide in abdomen. Color in alcohol light brown. Prostomium conical with palpode, eyespots present. Peristomium to seventh thoracic chaetiger with epithelium longitudinally striated, remaining segments smooth. Thorax with 11 chaetigers, with bilimbate capillaries in both rami first chaetiger biramous. Transition between thorax and abdomen marked by a slight reduction in the size of the first two abdominal segments (Fig. 2B). Thoracic and abdominal segments biannulated, notopodia lateral in first thoracic segments, in subsequent segments located dorsally (Fig. 2A). Lateral organs between notopodia and neuropodia throughout body, those of thoracic region closer to notopodium. In segments 12 and 13 the lateral organs protrude, just as abdominal ones. Genital pores not seen. Abdominal chaetigers 1-9 with notopodial lobes fused dorsally (Fig. 2B), each line of hooded hooks completely separated, with around 13 hooks per fascicle. Neuropodial lobes projected to dorsal region and separate ventrally (Fig. 2C), chaetal fascicles with 81 hooded hooks. Notopodial and neuropodial abdominal hooded hooks similar throughout, with long anterior shaft, bulbous node, indistinct constriction, developed shoulder, short hood, posterior shaft longer than anterior one, four rows of teeth above triangular main fang (Fig. 2D). Pygidium not examined.

Methyl green staining pattern. Peristomium and first seven chaetigers with light green, chaetiger 9–11 with moderate green, chaetiger 12th and pre-chaetal part of the 13th dark green (Fig. 2A). Abdominal chaetiger with two longitudinal bands stained with an intense green, disrupted by neuropodial lobes and lateral organs (Fig. 2B); with a longitudinal ventral line stained with a moderate green along the body (Fig. 2C).

####### Habitat.

Sediment characterized as sandy mud, muddy sand and sand with shell fragments, 21 to 55 m.

####### Distribution.

Only known for the type locality, Andaman Sea, Thailand.

####### Remarks.

In the description of *D. fauchaldi*, Green (2002) mentioned that the species had a tessellated epithelium up to chaetiger 4 or 5, hooded hooks with three lines of small teeth over the principal fang, and the ventral abdominal segments did not stain with methyl green. However the paratype reviewed has tessellated epithelium from the peristomium to segment 7, hooded hooks with four lines of small teeth over the principal fang, and methyl green staining revealed a longitudinal abdominal line with moderate green along ventral body. It is worth mention that in Green´s description, the legend of figure 23 B, F and G has a mistake, in figure B chaetiger 9-14, must actually be 11–16: in figure F chaetiger 10–15, must actually be 12–17, and in figure G chaetiger 10–14, must be 12–16.

###### Notodasus eibyejacobseni

Taxon classificationAnimaliaCapitellidaCapitellidae

(Green, 2002) comb. n.

Dodecaseta eibyejacobseni Green, 2002: 249-343, fig. 24A–G

####### Remarks.

The type material of *Dodecaseta eibyejacobseni* could not be review because it could not be located in the Phuket Marine Biological Center (Aungtonya, per. comm.). However, the original description of Green (2002) and the figure 24 A–G, provides evidence of morphological characters that are present in the genus *Notodasus*. Thus, we considered that this species needs be reassigned to the genus *Notodasus*.

Green (2002) states that she compared specimens of *N. eibyejacobseni* with specimens identified by Hartman (1947) as *Dasybranchus lumbricoides* Grube, 1878 (LACM-AHF n 2222,1451-42, n2170, 14913-F2676), from intertidal areas of southern California, lower California and other parts of western Mexico. Green found similarities between Hartman´s specimens and her own specimens from the BIOSHELF material. However, as *D. lumbricoides* was originally described from Pandanon Island (Philippines) there is a possibility that specimens from Thailand by Green and those from California and Mexico by Hartman do not belong to the same species as demonstrated below.

We reviewed some lots collected in California and identified by Olga Hartman and Kristian Fauchald as *D. lumbricoides*: LACM AHF POLY-N-1450-42, N1451-42; LACM AHF POLY-N 2221; LACM AHF POLY-N 1284/14884-F2637 and LACM AHF POLY-N 14905/F2663 (by O. Hartman), and LACM AHF POLY- N-1746 (by K. Fauchald). Specimens of the first four lots, all correspond to *Notodasus harrisae* García-Garza et al., 2009; specimen LACM AHF POLY-N 1284/14884-F2637 correspond to *Notodasus oraria* new combination; and specimen LACM AHF POLY-N 14905/F2663 correspond to *Dasybranchus platyceps* Hartman, 1947.

Ewing (1984) reported *D. lumbricoides* from the northern Gulf of Mexico, Florida however, based on his description, these specimens belong in the genus *Notodasus* but we do not have access to specimens in order to corroborate our observations.

## Supplementary Material

XML Treatment for Notodasus

XML Treatment for Notodasus oraria

XML Treatment for Notodasus fauchaldi

XML Treatment for Notodasus eibyejacobseni
